# Low-Temperature Loop-Mediated Isothermal Amplification Operating at Physiological Temperature

**DOI:** 10.3390/bios13030367

**Published:** 2023-03-10

**Authors:** Daehan Nam, Seokjoon Kim, Jung Ho Kim, Seungjin Lee, Daneub Kim, Jinseo Son, Doyeon Kim, Byung Seok Cha, Eun Sung Lee, Ki Soo Park

**Affiliations:** Department of Biological Engineering, College of Engineering, Konkuk University, Seoul 05029, Republic of Korea

**Keywords:** loop-mediated isothermal amplification, low-temperature, probe length, miRNA, molecular diagnostics, nucleic acid biomarkers

## Abstract

Loop-mediated isothermal amplification (LAMP) is one of the most widely used isothermal amplification technologies in molecular diagnostics. However, LAMP operates at a high temperature of 65 °C; thus, operating LAMP at a lower temperature is desirable to maximize its usefulness for on-site diagnosis. In this study, we propose a new version of LAMP, termed low-temperature LAMP, which operates at the physiological temperature of 37 °C. Low-temperature LAMP differs from conventional LAMP operating at 65 °C in terms of the concentrations of MgSO_4_ and deoxyribonucleoside triphosphates (dNTPs), as well as the lengths of DNA probes, which are crucial for the execution of low-temperature LAMP. Under the optimal conditions, the amplification efficiency of low-temperature LAMP is comparable to that of conventional LAMP. In addition, the ligation reaction at 37 °C, which is necessary to detect actual target nucleic acids, is combined without altering the temperature, enabling the identification of miR-21, a cancer-promoting oncogenic miRNA, with high sensitivity and selectivity. The method described in this paper does not require expensive DNA modifications or special additives and would facilitate the widespread application of LAMP in facility-limited or point-of-care settings, paving the way to improvements in other isothermal-amplification-based techniques.

## 1. Introduction

Various nucleic acid biomarkers, such as non-coding and viral RNAs, have been used for diagnosing cancers and infectious diseases [1,2,3]. The emergence of the novel coronavirus in 2019 and the associated economic and social damage have escalated the importance of nucleic acid amplification based detection technology [4,5,6]. The polymerase chain reaction (PCR) is a representative nucleic acid amplification based detection technology that is widely used for the sensitive and selective detection of target nucleic acid biomarkers [7]. However, PCR has severe drawbacks that hinder its application in facility-limited or point-of-care (POC) settings. First, it requires a lot of energy to raise the temperature to 95 °C for the denaturation of double-stranded DNA (dsDNA) [8]. Second, repeated thermal cycling steps (e.g., 40 cycles) are required; accordingly, specialized equipment is necessary [8,9].

To overcome these issues, isothermal amplification techniques operating at a constant temperature have been proposed as alternatives. Various isothermal amplification technologies, such as nucleic acid sequence based amplification (NASBA) [10], strand displacement amplification (SDA) [11], helicase-dependent amplification (HDA) [12], recombinase polymerase amplification (RPA) [13], rolling circle amplification (RCA) [14], the exponential amplification reaction (EXPAR) [15], and loop-mediated isothermal amplification (LAMP) [16], have been developed.

Since its discovery in 2000 [16], LAMP has been extensively used to analyze various nucleic acid biomarkers, such as DNA and RNA, in molecular diagnostics [17,18]. LAMP exhibits a high amplification efficiency [19,20], and using multiple DNA primers contributes to its enhanced specificity [19,20,21,22]. Furthermore, a single enzyme, *Bst* DNA polymerase, is required for the execution of LAMP, which decreases the overall assay cost, and is relatively stable against amplification inhibitors, leading to the detection of nucleic acid biomarkers, even without the extraction/purification of nucleic acids [19].

Despite its advantages, the LAMP method could be further optimized. For example, the reaction temperature, 60–65 °C, is relatively high; employing LAMP at a lower temperature that is closer to the physiological temperature (e.g., 37 °C) would facilitate its use for on-site diagnosis. In recent years, a version of LAMP has been implemented using phosphorothioated primers, which operates under the principle that phosphorothioate modification substantially decreases the melting temperature of phosphorothioate-phosphodiester dsDNA, thereby inducing more breathing at the termini of dsDNA [23]. Although this method exhibits enhanced amplification efficiency at a relatively low temperature of 40 °C, the requirement for expensive DNA modification (e.g., phosphorothioate) in both forward and backward inner primers substantially increases the overall analysis cost. Therefore, further research is required to develop low-cost and viable low-temperature LAMP alternatives to facilitate their on-site diagnosis.

In this study, we systematically investigated the optimal reaction conditions and DNA probe lengths to develop a new version of LAMP that operates at 37 °C. We compared the efficacy of our novel low-temperature LAMP with that of conventional LAMP, which operates at 60–65 °C. Furthermore, we evaluated its sensitivity and selectivity for detecting the target miRNA-21 in combination with the reaction catalyzed by SplintR ligase. This novel method was developed to provide low-cost and low-temperature diagnostic alternatives to facility-limited and/or point-of-care settings.

## 2. Materials and Methods

### 2.1. Reagents and Materials

The oligonucleotide sequences used in this study (Table S1) were purchased from Bionics (Seoul, Republic of Korea) and Integrated DNA Technologies (Coralville, IA, USA). *Bst* 2.0 DNA polymerase, 10× isothermal amplification buffer, *Bsu* DNA polymerase large fragment, 10× NEBuffer™ 2, SplintR ligase, 10× SplintR ligase reaction buffer, and dNTPs were purchased from New England Biolabs (NEB) (Ipswich, MA, USA). Klenow DNA polymerase exo-, phi29 DNA polymerase, 10× phi29 DNA polymerase buffer, MgSO_4_, and bovine serum albumin (BSA) were purchased from Enzynomics (Daejeon, Republic of Korea). Tween 20 was purchased from Sigma–Aldrich (St. Louis, MO, USA). Dithiothreitol (DTT) and SYBR Green I were purchased from Thermo Fisher Scientific (Waltham, MA, USA).

### 2.2. Conventional LAMP Assay

Conventional LAMP was performed according to the typical LAMP protocol using *Bst* 2.0 DNA polymerase (NEB) [24]. For the reaction, 1× isothermal amplification buffer (20 mM Tris-HCl, 10 mM (NH_4_)_2_SO_4_, 50 mM KCl, 2 mM MgSO_4_, and 0.1% Tween 20; pH 8.8 at 25 °C), 6 mM MgSO_4_ (total: 8 mM), 5.6 mM dNTPs (1.4 mM each), 0.5× SYBR Green I, 1.6 µM C-forward inner primer (FIP), 1.6 µM C-backward inner primer (BIP), 0.4 U/µL *Bst* 2.0 DNA polymerase, and various concentrations of C-stem–loop probe (SLP) were mixed. Nuclease-free water was added to bring the volume to 20 µL. The fluorescence signal was measured once every minute at 65 °C using a Gentier 96E real-time PCR system (Tianlong, Xi’an, China). The Ct value, which is the number of minutes required for the fluorescence signal to exceed the background fluorescence, was calculated. All experiments were performed in triplicate.

### 2.3. Optimizing the Reaction Composition of Low-Temperature LAMP

Unless otherwise stated, 1× isothermal amplification buffer (20 mM Tris-HCl, 10 mM (NH_4_)_2_SO_4_, 50 mM KCl, 2 mM MgSO_4_, and 0.1% Tween 20; pH 8.8 at 25 °C), 0.4 mM dNTPs (0.1 mM each), 0.5× SYBR Green I, 400 nM FIP-10|15, 400 nM BIP-10|15, 0.2 U/µL *Bst* 2.0 DNA polymerase, and 500 pM C22 were mixed, and the volume was increased to 20 µL with nuclease-free water. In a Gentier 96E real-time PCR system (Tianlong, Xi’an, China), the fluorescence signal was measured once every minute at 37 °C, and the Ct value, which is the number of minutes required for the fluorescence signal to exceed the background fluorescence, was calculated. All experiments were performed in triplicate.

### 2.4. Optimizing the DNA Probe of Low-Temperature LAMP

Unless otherwise stated, 1× isothermal amplification buffer (20 mM Tris-HCl, 10 mM (NH_4_)_2_SO_4_, 50 mM KCl, 2 mM MgSO_4_, and 0.1% Tween 20; pH 8.8 at 25 °C), 0.75 mM MgSO_4_ (total: 2.75 mM), 0.1 mM dNTPs (0.025 mM each), 2.5% Tween 20 (total: 2.6%), 0.5× SYBR Green I, 400 nM different FIP-#|# and BIP-#|# (Table S1), 0.2 U/µL *Bst* 2.0 DNA polymerase, and various concentrations of SLP were mixed, and the reaction volume was increased to 20 µL with nuclease-free water. The reactions were performed in accordance with the protocol described earlier, albeit at a temperature of only 37 °C.

### 2.5. Assay Validation in a Regular Heat Block

Unless otherwise stated, 1× isothermal amplification buffer (20 mM Tris-HCl, 10 mM (NH_4_)_2_SO_4_, 50 mM KCl, 2 mM MgSO_4_, and 0.1% Tween 20; pH 8.8 at 25 °C), 0.75 mM MgSO_4_ (total: 2.75 mM), 0.1 mM dNTPs (0.025 mM each), 0.5× SYBR Green I, 2.5% Tween 20 (total: 2.6%), 400 nM FIP-14|15, 400 nM BIP-10|13, 0.2 U/µL Bst 2.0 DNA polymerase, and 50 fM RL15 were mixed, and the volume was increased to 20 µL with nuclease-free water. The reactions were performed in a Thermo Shaker Incubator (Miulab, Hangzhou, China) at a temperature of 37 °C, and their fluorescence images were photographed every 5 minutes in an FAS-nano gel illuminator (Nippongenetics, Düren, Germany).

### 2.6. Low-Temperature LAMP for the Detection of miR-21

Unless otherwise stated, 1× SplintR ligase reaction buffer (50 mM Tris-HCl, 10 mM MgCl_2_, 1 mM ATP, and 10 mM DTT; pH 7.5 at 25 °C), 50 nM SLP-L, 50 nM SLP-R, 1.25 U/µL SplintR ligase, and various concentrations of miRNA were first mixed, and nuclease-free water was added to bring the volume to 20 µL. The reaction mixture was then incubated at 37 °C for 30 min to perform the ligation reaction. Subsequently, 1× isothermal amplification buffer (20 mM Tris-HCl, 10 mM (NH_4_)_2_SO_4_, 50 mM KCl, 2 mM MgSO_4_, and 0.1% Tween 20; pH 8.8 at 25 °C), 0.75 mM MgSO_4_ (total: 2.75 mM), 0.1 mM dNTPs (0.025 mM each), 0.5× SYBR Green I, 2.5% Tween 20 (total: 2.6%), 400 nM FIP-14|15, 400 nM BIP-10|13, 0.2 U/µL *Bst* 2.0 DNA polymerase, and 2 µL of ligation product were mixed, and nuclease-free water was added to increase the volume to 20 µL. The reactions were performed in accordance with the protocol described earlier, albeit at a temperature of only 37 °C.

## 3. Results and Discussion

### 3.1. Construction of Low-Temperature LAMP

**Figure 1:**
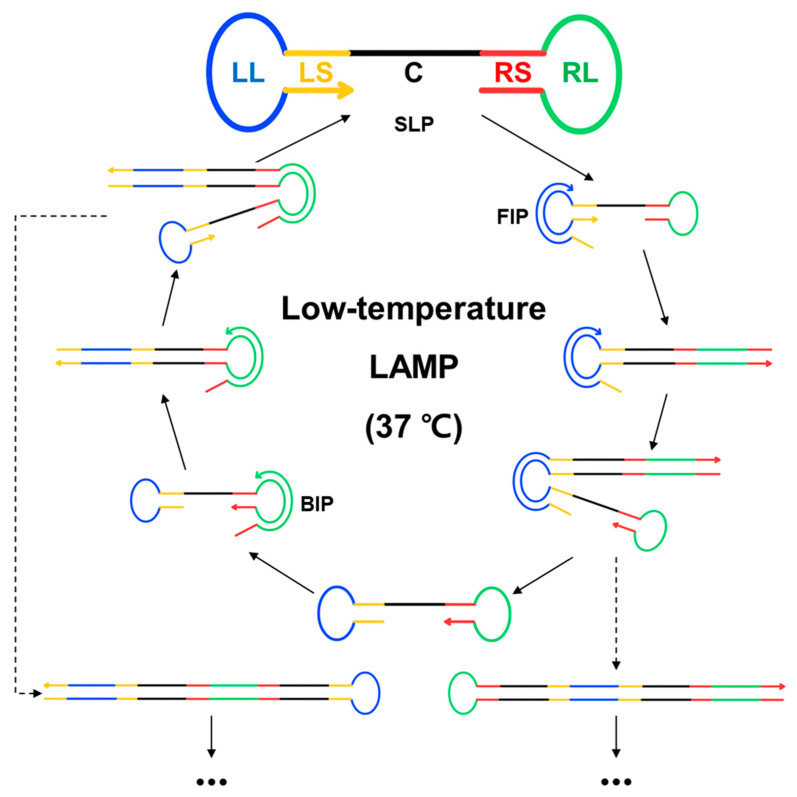
Schematic illustration of low-temperature LAMP, which operates at 37 °C, with the stem–loop probe (SLP) as the model DNA template. LAMP, loop-mediated isothermal amplification; LL, left loop; LS, left stem; C, center; RS, right stem; RL, right loop; BIP, backward inner primer; FIP, forward inner primer.

Figure 1 depicts a schematic illustration of low-temperature LAMP, where the stem–loop probe (SLP) is dumbbell-shaped and consists of five different regions: the left loop (blue, LL), left stem (yellow, LS), center (black, C), right stem (red, RS), and right loop (green, RL). 

Similar to the general workflow of conventional LAMP, *Bst* 2.0 DNA polymerase with strand displacement activity is used to initiate DNA synthesis with the help of specially designed DNA primers (FIP and BIP) that bind to SLP loop structures, which facilitates the repeated rounds of amplification by extending the loops and facilitating the additional annealing of primers. Consequently, long DNA products containing numerous repeats of the SLP sequence, connected with single-stranded loop regions in long concatemers, are formed at a reaction temperature of 37 °C. These products are monitored using SYBR Green I, which emits a highly enhanced fluorescence signal after intercalation into the dsDNA. 

By systematically investigating various reaction conditions and SLP lengths, we determined that the reaction conditions (e.g., the concentrations of Mg^2+^ and dNTPs) and the length of each part of the SLP (e.g., LS, RS, LL, and RL) are critical for the construction of low-temperature LAMP operating at 37 °C. As summarized in Table 1, the concentrations of Mg^2+^, dNTPs, and inner primers (FIP and BIP) were significantly lower in low-temperature LAMP than in conventional LAMP. Furthermore, the stem (LS and RS) and loop lengths (LL and RL) were shorter in low-temperature LAMP than in conventional LAMP. Concordantly, the SLP length was shorter in low-temperature LAMP. 

**Figure 2:**
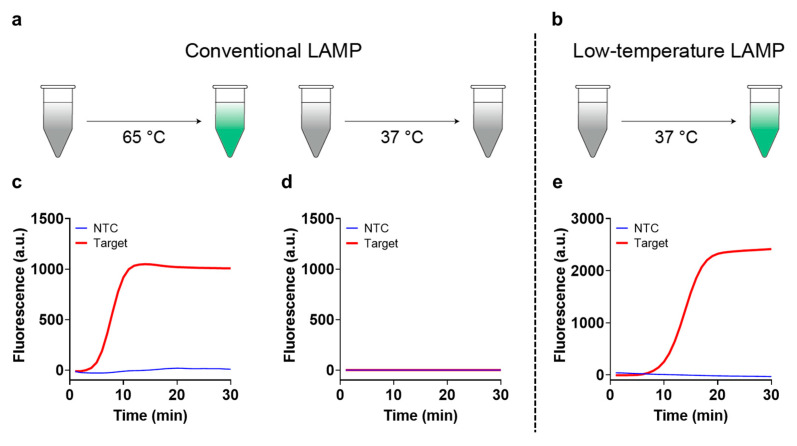
Comparison of conventional and low-temperature LAMPs. Schematic illustration describing the reaction results of the conventional (**a**) and low-temperature LAMP (**b**) methods at different temperatures (65 and 37 °C, respectively). The gray and green colors indicate the negligible and strong fluorescence signals of SYBR Green I, respectively. The amplification curves of the conventional LAMP reaction were obtained at (**c**) 65 °C and (**d**) 37 °C. NTC and Target indicate the absence and presence of C-SLP (50 pM), respectively. (**e**) The amplification curve of the low-temperature LAMP reaction obtained at 37 °C. NTC and Target indicate the absence and presence of RL15 (50 pM), respectively. All tests were performed with three technical replicates.

Under the optimal conditions of low-temperature and conventional LAMPs, we compared the amplification efficiencies of both systems (Figure 2a,b). The results in Figure 2c suggest that the reaction conditions and SLP length optimized for conventional LAMP enabled the effective amplification, generating a strong fluorescence signal as the amplification proceeded at 65 °C. However, when the temperature was decreased to 37 °C, the amplification did not occur, regardless of the presence of SLP (Figure 2d). The amplification was effectively executed at 37 °C only when the reaction conditions and SLP were optimized for a low temperature (Figure 2e), indicating that the low-temperature LAMP can be constructed without expensive modifications or special additives. 

### 3.2. Optimization of Reaction Conditions

**Figure 3:**
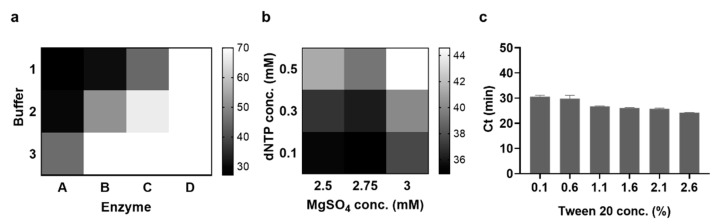
Optimization of reaction conditions for low-temperature LAMP. (**a**) Ct values according to the different combinations of DNA polymerase and reaction buffers. A, B, C, and D indicate *Bst* 2.0 DNA polymerase, Klenow DNA polymerase exo-, *Bsu* DNA polymerase large fragment, and phi29 DNA polymerase, respectively, while 1, 2, and 3 indicate 1× isothermal amplification buffer, 1× phi29 DNA polymerase buffer, and 1× NEBuffer™ 2, respectively. (**b**) Ct values according to the different concentrations of MgSO_4_ and dNTPs. The concentration of SLP (C22) was 5 pM. (**c**) Ct values according to the different concentrations of Tween 20. The concentrations of MgSO_4_ and dNTPs were 2.75 mM and 0.1 mM, respectively, and the concentration of SLP (C22) was 50 pM. All tests were performed with three technical replicates.

First, we tested various DNA polymerases and reaction buffers to execute low-temperature LAMP at 37 °C. Specifically, DNA polymerases with strand displacement activity (*Bst* 2.0 DNA polymerase, Klenow DNA polymerase exo-, *Bsu* DNA polymerase large fragment, and phi29 DNA polymerase) and their corresponding reaction buffers were selected, and the LAMP reaction was conducted at 37 °C with various combinations of DNA polymerase and reaction buffers. As shown in Figure 3a, the amplification was ineffective in the case of phi29 DNA polymerase (D), even with the combination of all three buffers (1–3). Meanwhile, *Bst* 2.0 DNA polymerase (A) demonstrated the most effective amplification efficiency, especially in the isothermal amplification buffer (1), as evidenced by the lowest Ct value and the strongest fluorescence signal at the endpoint (Figure S1). Therefore, subsequent experiments for low-temperature LAMP were performed using *Bst* 2.0 DNA polymerase in the isothermal amplification buffer. It should be noted that low-temperature LAMP worked well in a composition with a lower concentration of MgSO_4_ than in conventional LAMP.

Second, we investigated the effect of the MgSO_4_ and dNTP concentrations on low-temperature LAMP, with the assumption that the MgSO_4_ concentration is an important factor affecting the reaction rate. Contour plots were obtained in experiments with varying concentrations of MgSO_4_ (2–4 mM) and dNTPs (0.4–2.4 mM). As shown in Figure S2, MgSO_4_ (2–3.5 mM) and dNTPs (<0.5 mM) were effective for low-temperature LAMP. After determining the optimal concentration range, further optimization experiments were performed with specified concentrations of MgSO_4_ and dNTPs. The results in Figure 3b indicate that 2.75 mM MgSO_4_ and 0.1 mM dNTPs were optimal for effective low-temperature LAMP. Notably, the concentrations of Mg^2+^ and dNTPs were lower in the low-temperature LAMP than in the conventional LAMP (Table 1). We hypothesized that a lower Mg^2+^ concentration is necessary to avoid excessively strong hybridization at low temperatures and to direct the desirable amplification by minimizing the non-specific binding of DNA probes. Similarly, the concentration of dNTPs that chelate Mg^2+^ would also be lowered to balance the low concentration of Mg^2+^ in low-temperature LAMP [25,26].

Finally, we investigated the effects of Tween 20, BSA (Figure S3a), and dithiothreitol (DTT; Figure S3b), which are commonly used as additives to enhance the amplification efficiency of conventional LAMP [27], in low-temperature LAMP. As shown in Figure 3c, the Ct value decreased as the Tween 20 concentration increased, indicating an enhanced reaction rate. Although the reaction rate was expected to increase as the concentration of Tween 20 increased, to a certain level, we chose 2.6% Tween 20 for further experiments due to the bubble formation with a concentration over 2.6%, which can hinder reproducibility. In contrast, the concentrations of BSA and DTT did not affect the reaction rate (Figure S3); thus, these additives were not included in the subsequent experiments.

### 3.3. Optimization of Length of Each Part of SLP

**Figure 4:**
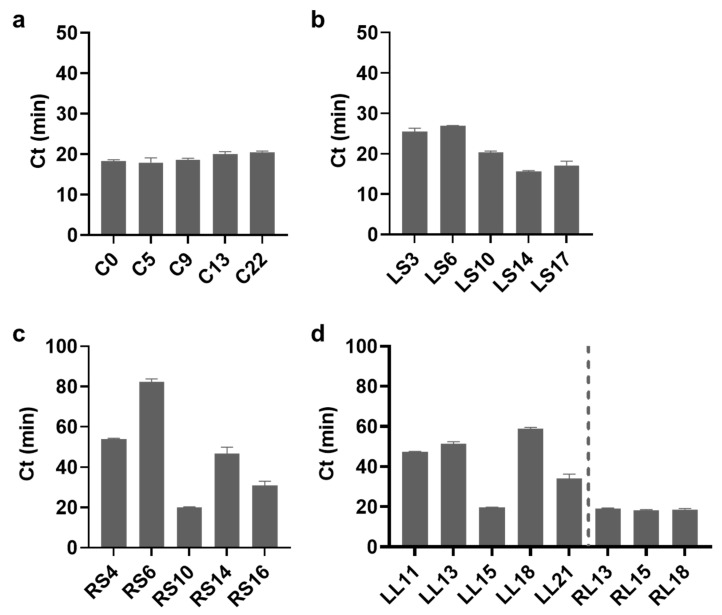
Optimization of the length on each part of the SLP for low-temperature LAMP. Ct values according to the different lengths of the (**a**) center (C), (**b**) left stem (LS), (**c**) right stem (RS), (**d**) left loop (LL), and right loop (RL) of the SLP. The grey dotted line is indicated to facilitate the distinction of LL and RL. The concentration of SLP was 50 pM. All tests were performed with three technical replicates.

Under optimal reaction conditions for low-temperature LAMP, the length of each part of the SLP was investigated to improve the amplification efficiency. First, the length of the C region was varied. As shown in Figure 4a, this did not significantly influence the amplification efficiency, implying that the low-temperature LAMP optimized in this study could be used to detect various target nucleic acids of different lengths. Next, the effect of the length of the LS and RS was evaluated; the 14- and 10-mer lengths exhibited the fastest reaction rates (Figure 4b,c). Finally, experiments conducted to verify the effects of the lengths of the LL and RL confirmed that the LL induced the fastest reaction speed at a 15-mer length, whereas the length of the RL did not affect the reaction speed (Figure 4d). Based on these results, SLPs with lengths of 14, 10, 15, and 15 mers for LS, RS, LL, and RL, respectively, were chosen for subsequent experiments. Notably, the SLP length in low-temperature LAMP was shorter than that in conventional LAMP (Table 1). We hypothesized that the SLP should be shortened to lower the hybridization strength to an appropriate level in low-temperature LAMP, which matched the results of the optimization of the reaction conditions. It should be noted that the optimal concentrations of MgSO_4_ and dNTPs that chelate Mg^2+^ may be different for probes of different lengths since the hybridization strength is dependent on both the hybridization length and the Mg^2+^ concentration. 

### 3.4. Optimization of Primer Length and Concentration

**Figure 5:**
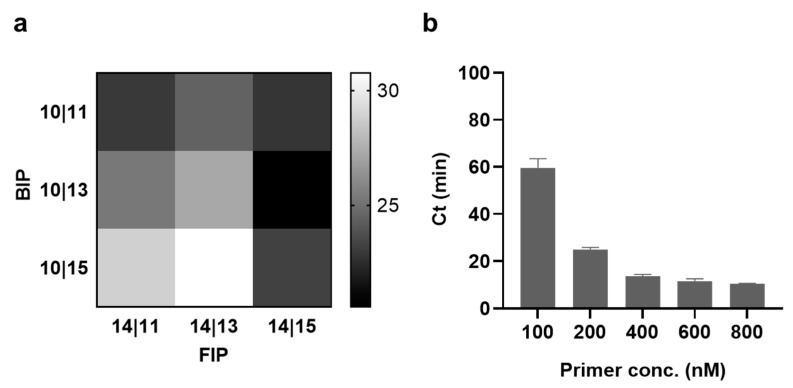
Optimization of hybridization length between primer and loop of SLP. (**a**) Ct values according to the different combinations of the forward inner primer (FIP) and backward inner primer (BIP) (stem hybridization length|loop hybridization length). The concentration of SLP (RL15) was 50 pM. (**b**) Ct values according to the different concentrations of primers. The concentration of RL15 was 50 pM. All tests were performed with three technical replicates.

We also investigated the effect of the binding length between the primer and loop of the SLP on the reaction rate by varying the length of the loop-binding region of the FIP and BIP. The two numbers (#|#) next to the FIP indicate the hybridization length of the FIP with the LS and LL, whereas those next to the BIP indicate the hybridization length of the BIP with the RS and RL, respectively (Table S1). Because the SLP stem lengths were optimized in Figure 4b,c, the hybridization lengths of both the FIP and BIP with the LS and RS of the SLP were determined to be 14 in the FIP and 10 in the BIP, respectively. As shown in Figure 5a, the optimal results were obtained when FIP-14|15 and BIP-10|13 were used, where the hybridization lengths of the FIP and BIP with the LL and RL of the SLP were 15 and 13, respectively. Furthermore, the concentrations of FIP-14|15 and BIP-10|13 were also optimized. The results in Figure 5b suggest that the Ct value decreased as the concentrations of the FIP and BIP increased, but no significant decrease was observed after 400 nM. Therefore, subsequent experiments were performed with a primer concentration of 400 nM.

### 3.5. Sensitivity of Low-Temperature LAMP

**Figure 6:**
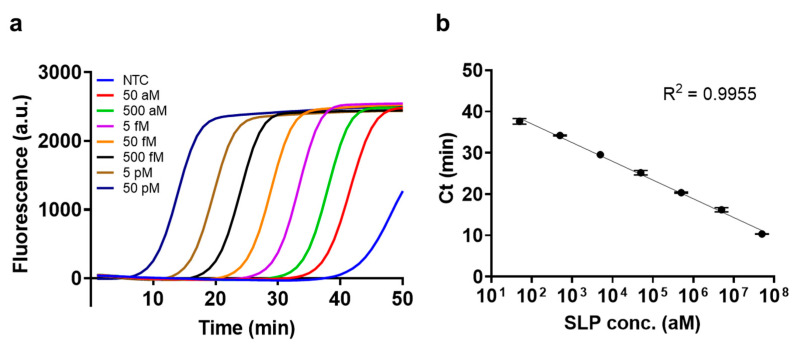
Sensitivity of low-temperature LAMP. (**a**) The amplification curves of low-temperature LAMP at different concentrations of SLP (RL15). (**b**) Standard curve of the Ct values at varying SLP concentrations. All tests were performed with three technical replicates.

After determining the optimized reaction conditions, SLP, and inner primers (FIP/BIP), the sensitivity of the low-temperature LAMP assay was investigated. The results in Figure 6 indicate that the Ct values decreased as the SLP concentration increased from 50 aM to 50 pM, exhibiting a high linear correlation coefficient (R^2^ = 0.9955) between the log concentration of SLP and the Ct value, and the detection limit was calculated to be 5 aM using the following formula: no template control (NTC)-3σ, where σ is the standard deviation of NTC. With the low-temperature LAMP, which operated at 37 °C, we obtained a high sensitivity at the aM level, comparable to that of conventional LAMP [28,29].

### 3.6. Assay Validation in a Regular Heat Block

We performed the low-temperature LAMP assay in a regular heat block. As shown in Figure S4, the fluorescence signals during low-temperature LAMP were negligible from 5 to 20 min in both NTC and SLP. However, the fluorescence signals started to increase at 25 min in the presence of SLP and became intensified at 30 min, which was not observed in the case of NTC. These results matched well with the amplification curve obtained in the presence of SLP (50 fM, Figure 6), proving that our assay can be reproduced without the need for qPCR equipment and is suitable for POC testing.

### 3.7. Application of Low-Temperature LAMP for the Detection of miRNA

**Figure 7:**
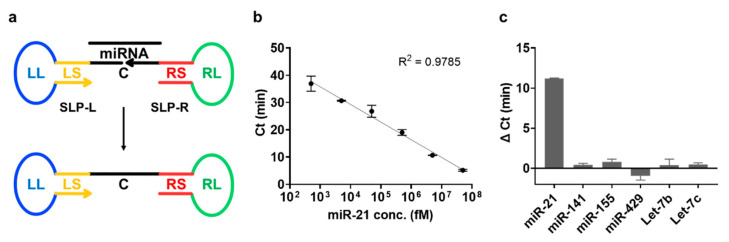
Detection of target miRNA using low-temperature LAMP in combination with a ligation reaction. (**a**) Schematic illustration of DNA ligation catalyzed by SplintR ligase for the detection of a target miRNA. (**b**) Standard curve of the Ct values at different miR-21 concentrations. (**c**) Specificity of low-temperature LAMP for the detection of the target miRNA. The Δ Ct values refer to the difference between the Ct value of each miRNA and the Ct value of NTC. The concentration of miRNA was 500 pM. All tests were performed with three technical replicates.

We attempted to detect miRNA to demonstrate the applicability of the proposed low-temperature LAMP method. Similar to conventional LAMP for the detection of miRNA, the ligation reaction catalyzed by SplintR ligase was employed before the execution of LAMP, as described in Figure 7a. The presence of the target miRNA resulted in a ligation between the left stem–loop probe(SLP-L) and the right stem–loop probe (SLP-R) by SplintR ligase, which formed a dumbbell-like structure that could be used as a template for low-temperature LAMP. Notably, the entire process, including ligation and low-temperature LAMP, could be operated at 37 °C, facilitating the on-site diagnosis of various nucleic acid biomarkers. As a proof of concept, we chose miR-21 as a model target miRNA to be analyzed using the proposed strategy. The results in Figure 7b and Figure S5 suggest that miR-21, whose concentration ranges from 500 fM to 50 nM, was successfully analyzed, with a high correlation coefficient (R^2^ = 0.9785), and the detection limit calculated using NTC-3σ was 130 fM. In addition, the specificity of low-temperature LAMP was confirmed by evaluating the detection of other miRNAs, such as miR-141, miR-155, miR-429, let-7b, and let-7c. As shown in Figure 7c, only the presence of miR-21 generated a significant Δ Ct value due to the reduced Ct values, indicating the high selectivity, without any cross-reactivity, of the proposed system.

## 4. Conclusions

In this study, we developed a low-temperature LAMP that operates at 37 °C by systematically investigating the reaction conditions and the lengths of DNA probes, without expensive DNA modifications or special additives. Importantly, the attomolar sensitivity achieved with low-temperature LAMP was comparable to that of conventional LAMP, but low-temperature LAMP, compared to conventional LAMP functioning at 65 °C, is better suited for applications in facility-limited or POC settings. In addition, it can be applied to detect target miRNAs in combination with ligation reactions. However, a loss of sensitivity was observed during ligation compared to when only low-temperature LAMP was performed; thus, increasing the ligation efficiency is required to compensate for the loss of sensitivity. Moreover, further research is required to demonstrate the versatile applicability of this technique by targeting various nucleic acid biomarkers. Nonetheless, we believe this technique, which operates at a physiological temperature, could enable the development of a detection system, even without an incubator for temperature maintenance, and will provide guidelines for improving other isothermal amplification techniques.

## Figures and Tables

**Table 1:** Comparison of conventional and low-temperature LAMPs in terms of reaction components, conditions, and SLP lengths. The reaction conditions of conventional LAMP were adopted from the typical LAMP protocol using *Bst* 2.0 DNA polymerase (NEB) [24].

	Conventional LAMP	Low-Temperature LAMP
Buffer	1× isothermal amplification buffer	1× isothermal amplification buffer
Temperature	65 °C	37 °C
Mg^2+^	8 mM	2.75 mM
dNTPs	5.6 mM (1.4 mM each)	0.1 mM (0.025 mM each)
Inner primer (FIP/BIP) concentration	1.6 µM	400 nM
Stem lengths (LS and RS)	21 mers and 20 mers	14 mers and 10 mers
Loop lengths (LL and RL)	42 mers and 40 mers	15 mers and 15 mers
SLP length	175 mers	78 mers

## Data Availability

Data will be made available on request.

## Supplementary Materials

The following supporting information can be downloaded at https://www.mdpi.com/article/10.3390/bios13030367/s1, Figure S1: Amplification curve according to the different combinations of DNA polymerases and reaction buffers; Figure S2: Contour plots of Ct values as functions of MgSO_4_ and dNTP concentrations; Figure S3: Ct values according to the different concentrations of (a) BSA and (b) DTT; Figure S4: Fluorescence images taken at 5-minute intervals in a regular heat block; Figure S5: Amplification curves of low-temperature LAMP in combination with the ligation reaction at different concentrations of miR-21; Table S1: Sequences of oligonucleotides used in this study [30].
