# Supplementary material for: Low-Temperature Loop-Mediated Isothermal Amplification Operating at Physiological Temperature
Source: Biosensors (Basel). 2023 Mar 10;13(3):367. doi: 10.3390/bios13030367 (PMC10046060; doi:10.3390/bios13030367)
Supplement: Supplementary file 1 [file biosensors-13-00367-s001.zip › biosensors-2218740-supplementary.pdf]

Supplementary Information

Low-Temperature Loop-Mediated Isothermal Amplification Operating at Physiological Temperature

Daehan Nam,[#] Seokjoon Kim,[#] Jung Ho Kim, Seungjin Lee, Daneub Kim, Jinseo Son, Doyeon Kim, Byung Seok Cha, Eun Sung Lee and Ki Soo Park[*]

Department of Biological Engineering, College of Engineering, Konkuk University, Seoul 05029, Republic of Korea

# The authors contributed equally.
* Correspondence: akdong486@konkuk.ac.kr (K.S. Park); Phone: +82-2-450-3742; Fax: +82-2-450-3742.

Table S1. Sequences of oligonucleotides used in this study.

Name	Sequence (5′→3′ direction)	Primers*
	SLP to evaluate the effect of center (C) length	
C22	CCTGAGTAGAGTAATGCCGATAGTTTCTACTCAGGTCAACATCAGTCTGATAA-<br>GCTACACACTGGAACTATGGTAATCGTCATTCCAGTGTG	FIP-10|15<br>BIP-10|15
C13	CCTGAGTAGAGTAATGCCGATAGTTTCTACTCAGGGTCTGATAAGCTACACAC-<br>TGGAACTATGGTAATCGTCATTCCAGTGTG	FIP-10|15<br>BIP-10|15
C9	CCTGAGTAGAGTAATGCCGATAGTTTCTACTCAGGGTCTGATAACACACTG-<br>GAACTATGGTAATCGTCATTCCAGTGTG	FIP-10|15<br>BIP-10|15
C5	CCTGAGTAGAGTAATGCCGATAGTTTCTACTCAGGGATAACACACTGGAAC-<br>TATGGTAATCGTCATTCCAGTGTG	FIP-10|15<br>BIP-10|15
C0	CCTGAGTAGAGTAATGCCGATAGTTTCTACTCAGGCACACTGGAACTATGG-<br>TAATCGTCATTCCAGTGTG	FIP-10|15<br>BIP-10|15
	SLP to evaluate the effect of left stem (LS) length	
LS3	CCTGAGTAGAGTAATGCCGATAGTTTCTACTCAGGCTGCTATGG-<br>TAATCGTCACAG	FIP-3|15<br>BIP-10|15
LS6	CCTGAGTAGAGTAATGCCGATAGTTTCTACTCAGGCTGGAACTATGG-<br>TAATCGTCATTCCAG	FIP-6|15<br>BIP-10|15
LS10<br>(C0)	CCTGAGTAGAGTAATGCCGATAGTTTCTACTCAGGCACACTGGAACTATGG-<br>TAATCGTCATTCCAGTGTG	FIP-10|15<br>BIP-10|15
LS14	CCTGAGTAGAGTAATGCCGATAGTTTCTACTCAGGCACACTGGAAGCAAC-<br>TATGGTAATCGTCATTGCTTCCAGTGTG	FIP-14|15<br>BIP-10|15
LS17	CCTGAGTAGAGTAATGCCGATAGTTTCTACTCAGGCACACTGGAAGCAAAGAC-<br>TATGGTAATCGTCATCTTTGCTTCCAGTGTG	FIP-17|15<br>BIP-10|15
	SLP to evaluate the effect of right stem (RS) length	
RS4	CCTGGTAATGCCGATAGTTCAGGCACACTGGAAGCAACTATGGTAATCGTCATT-<br>GCTTCCAGTGTG	FIP-14|15<br>BIP-4|15
RS6	CCTGAGGTAATGCCGATAGTTCTCAGGCACACTGGAAGCAACTATGG-<br>TAATCGTCATTGCTTCCAGTGTG	FIP-14|15<br>BIP-6|15
RS10<br>(LS14)	CCTGAGTAGAGTAATGCCGATAGTTTCTACTCAGGCACACTGGAAGCAAC-<br>TATGGTAATCGTCATTGCTTCCAGTGTG	FIP-14|15<br>BIP-10|15
RS14	CCTGAGTAGAGGTTGTAATGCCGATAGTTAACCTCTACTCAGGCACACTGGAA-<br>GCAACTATGGTAATCGTCATTGCTTCCAGTGTG	FIP-14|15<br>BIP-14|15
RS16	CCTGAGTAGAGGTTGAGTAATGCCGATAGTTTCAACCTCTACTCAGGCACACTG-<br>GAAGCAACTATGGTAATCGTCATTGCTTCCAGTGTG	FIP-14|15<br>BIP-16|15
	SLP to evaluate the effect of left loop (LL) or right loop (RL) length	
LL11	CCTGAGTAGAGTAATGCCGATAGTTTCTACTCAGGCACACTGGAA-<br>GCAAGGTAATCGTCATTGCTTCCAGTGTG	FIP-14|11<br>BIP-10|15
LL13	CCTGAGTAGAGTAATGCCGATAGTTTCTACTCAGGCACACTGGAAGCAAATGG-<br>TAATCGTCATTGCTTCCAGTGTG	FIP-14|13<br>BIP-10|15
LL15<br>(LS14, RS10)	CCTGAGTAGAGTAATGCCGATAGTTTCTACTCAGGCACACTGGAAGCAAC-<br>TATGGTAATCGTCATTGCTTCCAGTGTG	FIP-14|15<br>BIP-10|15
LL18	CCTGAGTAGAGTAATGCCGATAGTTTCTACTCAGGCACACTGGAAGCAAGAAC-<br>TATGGTAATCGTCATTGCTTCCAGTGTG	FIP-14|18<br>BIP-10|15
LL21	CCTGAGTAGAGTAATGCCGATAGTTTCTACTCAGGCACACTGGAAGCAAGTT-<br>GAACTATGGTAATCGTCATTGCTTCCAGTGTG	FIP-14|21<br>BIP-10|15
RL13	CCTGAGTAGAGTAATGCCGATAGTCTACTCAGGCACACTGGAAGCAACTATGG-<br>TAATCGTCATTGCTTCCAGTGTG	FIP-14|15<br>BIP-10|13
RL15 (LS14, RS10,<br>LL15)	CCTGAGTAGAGTAATGCCGATAGTTTCTACTCAGGCACACTGGAAGCAAC-<br>TATGGTAATCGTCATTGCTTCCAGTGTG	FIP-14|15<br>BIP-10|15
RL18	CCTGAGTAGAGTAATGCCGATAGTTCTGTCTACTCAGGCACACTGGAAGCAAC-<br>TATGGTAATCGTCATTGCTTCCAGTGTG	FIP-14|15<br>BIP-10|18
	FIP-# (Left stem hybridization length) | # (Left loop hybridization length)	
FIP-3|15	CTGTGACGATTACCATAG	
FIP-6|15	CTGGAATGACGATTACCATAG	
FIP-10|15	CACACTGGAATGACGATTACCATAG	
FIP-14|15	CACACTGGAAGCAATGACGATTACCATAG	
FIP-17|15	CACACTGGAAGCAAAGATGACGATTACCATAG	
FIP-14|11	CACACTGGAAGCAATGACGATTACC	
FIP-14|13	CACACTGGAAGCAATGACGATTACCAT	

FIP-14|18	CACACTGGAAGCAATGACGATTACCATAGTTC
FIP-14|21	CACACTGGAAGCAATGACGATTACCATAGTTCAAC
BIP-# (Right stem hybridization length) | # (Right loop hybridization length)	
BIP-4|15	CCTGGTAATGCCGATAGTT
BIP-6|15	CCTGAGGTAATGCCGATAGTT
BIP-10|15	CCTGAGTAGAGTAATGCCGATAGTT
BIP-14|15	CCTGAGTAGAGGTTGTAATGCCGATAGTT
BIP-16|15	CCTGAGTAGAGGTTGAGTAATGCCGATAGTT
BIP-10|11	CCTGAGTAGAGTAATGCCGAT
BIP-10|13	CCTGAGTAGAGTAATGCCGATAG
BIP-10|18	CCTGAGTAGAGTAATGCCGATAGTTCTG
Low-temperature LAMP for the detection of miRNA	
SLP-L	Phosphate-CTGATAAGCTACACACTGGAAGCAACTATGGTAATCGTCATTGCTTCCAGTGTG
SLP-R	CCTGAGTAGAGTAATGCCGATAGTTTCTACTCAGGTCAACATCAGT
miR-21	UAGCUUAUCAGACUGAUGUUGA
miR-141	UAACACUGUCUGGUA AAGAUGG
miR-155	UUAAUGCUAAUCGUGAUAGGGGUU
miR-429	UAAUACUGUCUGGUA AAACCGU
Let-7b	UGAGGUAGUAGGUUGUGUGGUU
Let-7c	UGAGGUAGUAGGUUGUAUGGUU
Conventional LAMP [1]	
C-SLP	GCTCCTCCACACGCTTCCATTGTATCCACCGTAGCCAG-TCTTAAGGTGGGCTGCGTGGTGATGGAAGCGTGTGGAGGAGCGAACCATTGGTATCGGACGTTTATGGGGATGGCCAACGCAGTTGATCAGTCCGCAGCACGTCAAACCGTATGTCCCATCCCCA-TAAACGTCCGAT
C-FIP	ATCGGACGTTTATGGGGATGG GACATACGGTTTGACGTGCTG
C-BIP	GCTCCTCCACACGCTTCCAT TGTATCCACCGTAGCCAGTC

Blue: left loop (LL); yellow: left stem (LS); black: center (C); red: right stem (RS); green: right loop (RL). The underlined and non-underlined sequences in the same color can hybridize. The names in parentheses indicate the same oligonucleotide. *Primers (FIP/BIP) used to perform low-temperature LAMP.

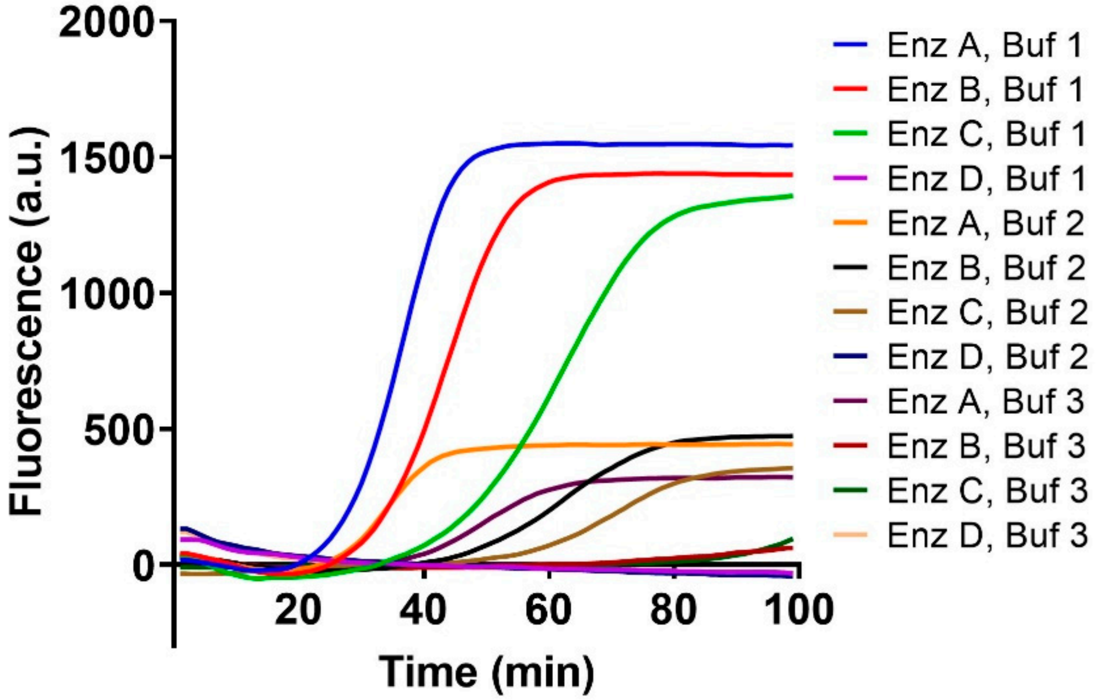

Figure S1. Amplification curve according to the different combinations of DNA polymerases and reaction buffers. A, B, C, and D indicate Bst 2.0 DNA polymerase, Klenow DNA polymerase exo-, Bsu DNA polymerase large fragment, and phi29 DNA polymerase, respectively, whereas 1, 2, and 3 indicate 1× isothermal amplification buffer, 1× phi29 DNA polymerase buffer, and 1× NEBuffer™ 2, respectively. All tests were performed with three technical replicates.

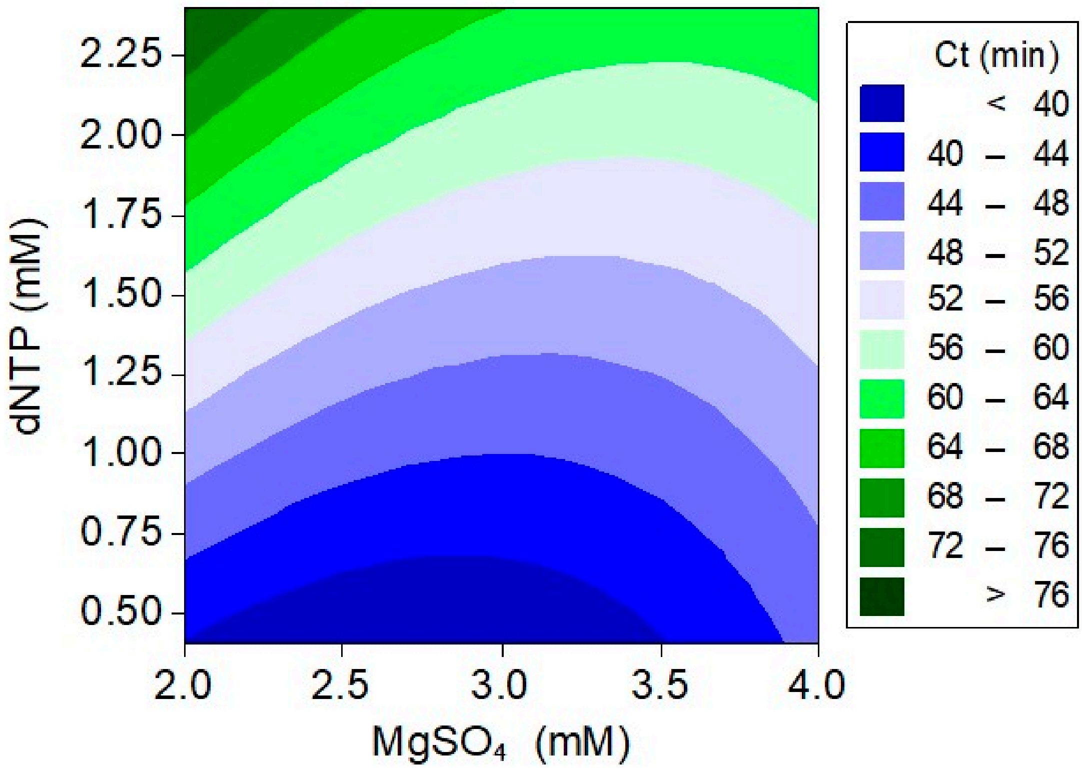

Figure S2. Contour plots of Ct value as a function of MgSO4 and dNTPs concentrations. The concentration of SLP (C22) used was 5 pM. All tests were performed with three technical replicates.

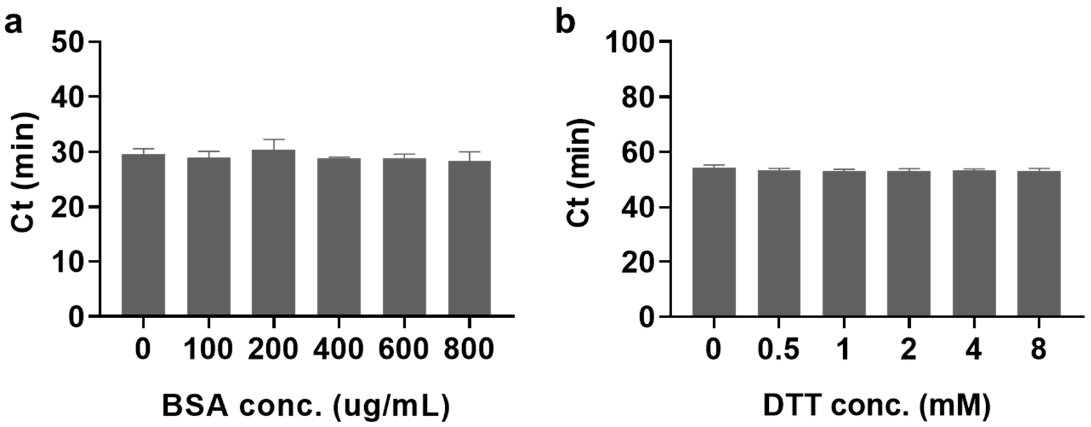

Figure S3. Ct value according to the different concentrations of (a) BSA and (b) DTT. In (a) and (b), the concentration of SLP (C22) used was 50 pM and 5 pM, respectively. All tests were performed with three technical replicates.

.

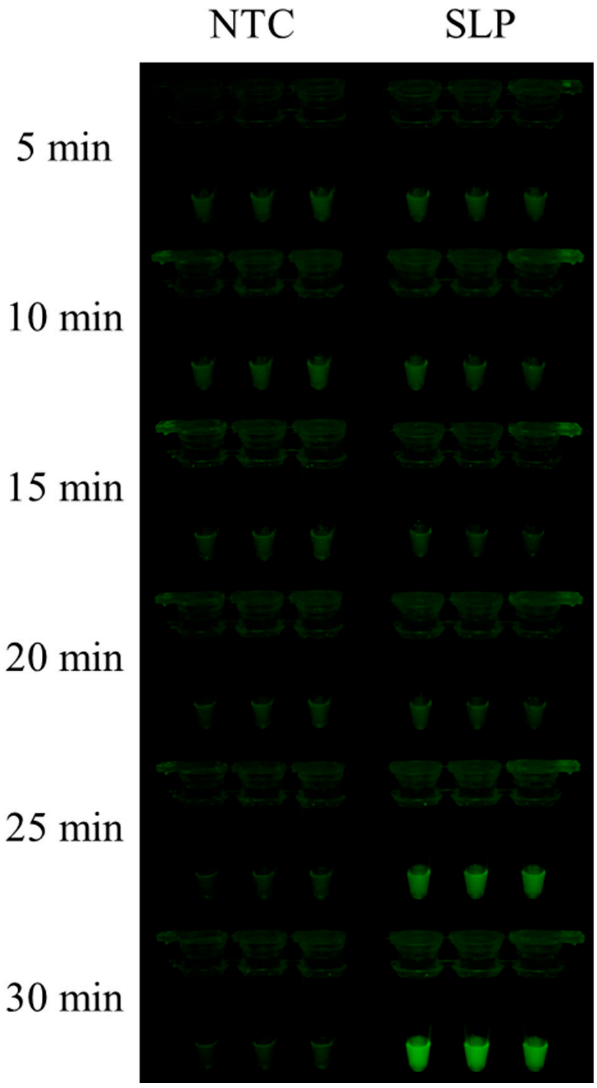

Figure S4. Fluorescence images taken at 5-minute intervals in a regular heat block. NTC indicates no template control. The concentration of SLP (RL15) used was 50 fM.

.

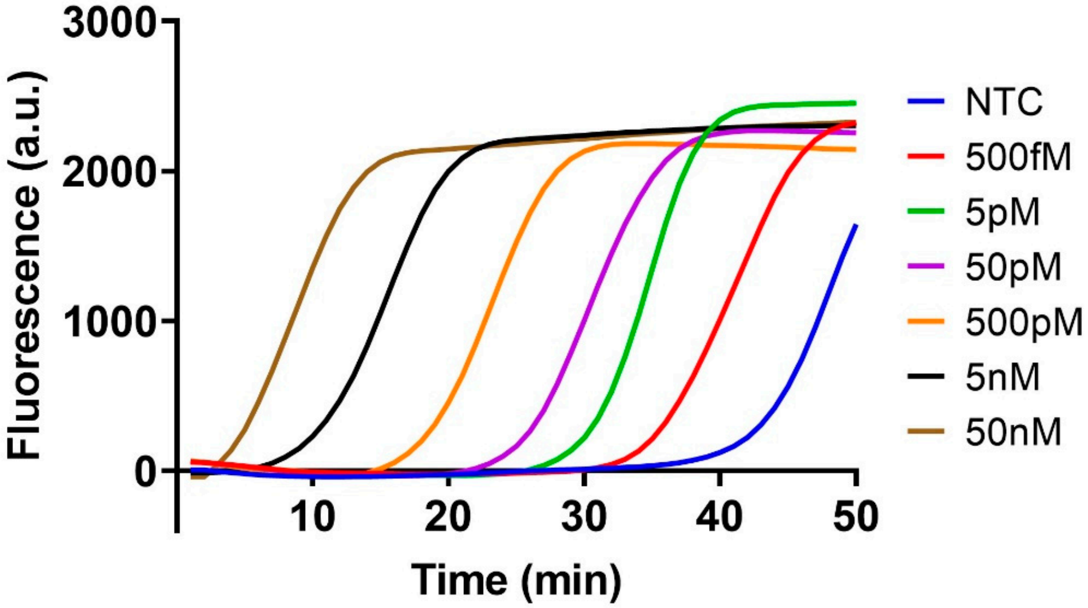

Figure S5. Amplification curves of the low-temperature LAMP in combination with ligation reaction at different concentrations of miR-21. NTC indicates no template control. All tests were performed with three technical replicates.

Reference

1. Sheikh, N.; Kumar, S.; Sharma, H.K.; Bhagyawant, S.S.; Thavaselvam, D. Development of a Rapid and Sensitive Colorimetric Loop-Mediated Isothermal Amplification Assay: A Novel Technology for the Detection of Coxiella Burnetii From Minimally Processed Clinical Samples. *Front. Cell. Infect. Microbiol.* **2020**, *10*, 127, doi:10.3389/fcimb.2020.00127.
